# Management of patients with prior lumbar fusion: a cross-sectional survey of Veterans Affairs chiropractors’ attitudes, beliefs, and practices

**DOI:** 10.1186/s12998-020-00322-9

**Published:** 2020-06-19

**Authors:** Clinton J. Daniels, Jordan A. Gliedt, Pradeep Suri, Edward M. Bednarz, Anthony J. Lisi

**Affiliations:** 1 Rehabilitation Care Services, VA Puget Sound Health Care System, 9600 Veterans Drive, Tacoma, WA 98493 USA; 2 Department of Neurosurgery, Medical College of Wisconsin, 9200 W. Wisconsin Ave., Milwaukee, WI 53226 USA; 3 Seattle Epidemiologic Research and Information Center, VA Puget Sound Health Care System, 1660 S Columbian Way, Seattle, WA 98108 USA; 4 Department of Rehabilitation Medicine, University of Washington, 325 9th Ave, Seattle, WA 98104 USA; 5 Chiropractic Service Chief, VA Connecticut Health Care System, 950 Campbell Ave, West Haven, CT 06516 USA

**Keywords:** Chiropractic, Manipulation, Postoperative periods, Spinal fusion, Surgical procedures, Operative

## Abstract

**Background:**

Little is known about the preferred treatment strategies of chiropractors in managing low back pain patients with prior lumbar fusions. There are several case reports which describe chiropractic care following surgical intervention, but there are no cohort or experimental studies published. Therefore, we sought to examine self-reported management approaches and practice patterns related to the management of patients with prior surgical lumbar fusion, among United States Veterans Affairs (VA) chiropractors.

**Methods:**

An electronic survey was administered nationwide to all chiropractors providing clinical care within VA. Questions were informed by a prior survey and piloted on a sample of chiropractors external to VA. Statistical analysis included respondent background information, and quantitative analysis of chiropractic referral patterns and practices. This survey collect information on 1) provider demographics, 2) VA referral patterns, and 3) attitudes, beliefs, practices and interventions utilized by VA chiropractors to manage patients with a history of surgical lumbar fusion.

**Results:**

The survey response rate was 46.3% (62/134). The respondents were broadly representative of VA chiropractic providers in age, gender, and years in practice. The majority of respondents (90.3%) reported seeing at least 1 post-fusion patient in the past month. The most common therapeutic approaches utilized by VA chiropractors were healthy lifestyle advice (94.9%), pain education (89.8%), exercise prescription (88.1%), stretching (66.1%) and soft tissue manual therapies (62.7%). A relatively smaller proportion described always or frequently incorporating lumbar (16.9%), thoracic (57.6%) or pelvic (39.0%) spinal manipulation.

**Conclusion:**

This survey provides preliminary data on VA chiropractic services in the management of patients with prior lumbar fusion. These patients are often seen by VA chiropractors, and our findings support the need for further study to advance understanding of interventions utilized by chiropractors in this patient population.

## Background

Low back pain (LBP) causes more disability globally than any other health condition [1]. In the United States (US), lumbar spinal fusion surgeries are commonly performed procedures for LBP and lumbar spinal disorders. National US data from the online Health Care Utilization Project, sponsored by the Agency for Healthcare Research and Quality, show the annual number of fusion operations (all indications and spinal levels) has increased from about 61,000 in 1993 to over 450,000 in 2013 [2, 3]. There was a 62.3% increase in the volume of elective lumbar fusion from 2004 to 2015 [4]. Following spine surgical procedures, up to 61% of patients continue to experience chronic spinal pain, though the frequency of chronic postoperative pain after lumbar fusion is not known [5–7].

Chiropractic treatments have been shown to be effective for LBP [8–12]. The American College of Physicians clinical practice guidelines recommends components of chiropractic care, such as spinal manipulation and exercise, for the management of acute and chronic LBP [13]. Chiropractic care is commonly utilized in the US, with approximately 190 million adult patient visits annually [14, 15], and services are covered by most public and commercial insurers [16]. As of 2004, chiropractic care has been included as a standard benefit in the Department of Veterans Affairs (VA) healthcare system, with overall use growing approximately 18% per year since then [17].

The reported point prevalence of postsurgical patients in US chiropractic clinics ranges from 2.3–12% [18–20]. Several case reports describe clinical improvements with chiropractic care in patients with postsurgical lumbar spine pain [21–27]. There are no cohort studies or experimental designs reporting on the outcomes of care provided by chiropractors in patients with prior lumbar fusion [28]. A recent VA research agenda from a state-of-the-art research conference on non-pharmacological care of chronic musculoskeletal conditions identified postoperative spine pain as a research priority for manual therapies, including manipulation and massage [29]. Though we are unaware of any data on the prevalence of postsurgical spine patients seen in VA chiropractic clinics, or the characteristics of care delivered.

A survey has been published describing typical clinical practices among physical therapists for patients with LBP and a prior history of lumbar spinal fusion [30], but no such surveys exist which describe the typical postsurgical clinical practices of chiropractors. An evaluation of current chiropractic practice is required to understand the present use of chiropractic services in VA and inform future research. We conducted a survey among chiropractic providers working within VA to characterize 1) provider demographics, 2) VA referral patterns to on-site chiropractic clinics for postoperative fusion patients, and 3) to identify trends and characteristics of attitudes, beliefs, practices, and interventions utilized by VA Doctors of Chiropractic (DC).

## Methods

### Design, setting, and methods

This was a self-administered cross-sectional survey following best-practices frameworks established by Burns [31], Drauglis [32], and Kelly [33]. Strengthening the Reporting of Observational Studies in Epidemiology (STROBE) guidelines have been followed for reporting of this research [34]. Ethical approval was provided through the VA Puget Sound Health Care System Institutional Review Board, MIRB#01560. Invitations with an information sheet and instructions on access to the survey (with hyperlink) were sent to the email addresses of all DCs working in a clinical setting in VA system.

Study data were collected and managed using REDCap electronic data capture tools hosted at VA Puget Sound Health Care System [35, 36]. The survey tool was administered and stored in the VA Informatics and Computing Infrastructure (VINCI), behind a VA firewall, with all information collected stored on the secure VA network with access restricted to designated study staff.

### Questionnaire development

Survey topics and questions were modeled on a recent cross-sectional study investigating United Kingdom physiotherapy practices related to lumbar spine fusion for non-Veterans [30], with modifications made for relevancy to VA DCs and chiropractic clinical practice. Completion of the survey was voluntary, anonymous, and Internet Protocol addresses were not recorded. No incentives were offered to respondents. Eligible DCs were invited to participate and were provided a participant information sheet describing the study, and potential risks and benefits of participation. Participant submission of the survey served as implied consent. The survey instrument was comprised of a combination of one free-text question, twenty-nine multiple-choice questions and a matrix-table with twenty-two 5-point Likert scale items with options of *always, frequently, sometimes, rarely and never*. Throughout the survey, respondents were given the opportunity to write-in free-text answers for further clarification or comment. The survey tool was piloted in a group of non-VA DCs (*n* = 8) and was revised to improve clarity based on the feedback provided, prior to administration to VA DCs. Piloting DCs reported an approximate survey completion time of 10 min.

The first section of the survey tool collected provider demographic information including age, gender, employment status, VA and chiropractic experience in years, hospital training, diplomate training, supplementary academic or professional degrees, and academic appointments. The second section collected information on the referral sources for patients with prior fusion, reasons for referral, average prevalence per month of referrals received and patients treated. The last section of the survey collected information on management and practice patterns, approach to care, imaging preferences, utilization of patient-reported outcome measures, and interdisciplinary communication. Providers were asked to answer questions related to the timeframe following surgical intervention in which they would be comfortable performing high-velocity low-amplitude manipulation to the thoracic, pelvic, or lumbar regions, and their expectation for dosage of chiropractic sessions typically needed to reach maximum therapeutic benefit (MTB).

### Participants and recruitment

In June–July 2018, all one-hundred thirty-four practicing chiropractic providers working within VA nationwide were contacted electronically and invited to participate through their VA e-mail. Reminders were sent at 3 weeks and 6 weeks after the initial invitation, if needed. Participants included practicing DCs employed by VA, chiropractic residents, “without compensation” academic affiliate DCs, and fee-basis consultant chiropractors working on VA campuses. Providers delivering care in the community which was purchased by VA were not surveyed. A list of all VA chiropractic site locations is available publicly online [37]. The individual providers were contacted from a complete list of email addresses for all chiropractors in VA system, which was maintained by the VA Chiropractic Service Line.

### Statistical analysis

All analyses were descriptive. We calculated frequencies and proportions for categorical variables, and means and standard deviations (SDs) for continuous variables. Analyses were conducted in Microsoft Excel 2016. All respondents that completed greater than 75% of the questions were included, and any missing answers were excluded from the analysis.

## Results

### Baseline characteristics of VA chiropractic study participants

Sixty-two (46.3%) of the 134 invited DCs responded. None completed less than 75% of the questions, and thus all 62 were included. The majority of respondents were male (n = 50, 80.6%) and the mean age was 44.5 years (SD 10.6 years). Most responding providers had substantial clinical experience with more than 50% having practiced for greater than 14 years (n = 36, 58.1%). The majority of respondents had been employed by VA for less than 6 years (n = 46, 74.2%) (Table 1).

**Table 1:** Baseline Characteristics of Study Participants, Veterans Affairs (VA) Chiropractors (n = 62)

Characteristic	Mean (SD) or Number (%)
Age (years)	44.5, SD 10.6
Sex	
Male	50 (80.6)
Female	12 (19.4)
Employment Status	
Salaried	56 (90.3)
Fee-Basis Consultant	3 (4.8)
“Without Compensation” Appointment	1 (1.6)
Resident	2 (3.2)
Years in Chiropractic Clinical Practice	
≤ 3	7 (11.3)
4–6	6 (9.7)
7–10	10 (16.1)
11–13	3 (4.8)
14–20	15 (24.2)
≥ 21	21 (33.9)
Years employed with VA	
≤ 3	27 (43.5)
4–6	19 (30.6)
7–10	5 (8.1)
11–13	7 (11.3)
14–20	4 (6.5)
≥ 21	0
Department of Chiropractic Clinic	
Pain Management	8 (12.9)
Physical Medicine and Rehabilitation	37 (59.7)
Primary Care	5 (8.1)
Other	12 (19.4)
Post-Graduate Degree (other than DC, missing = 1)	
Acupuncture	8 (12.9)
Doctor of Physical Therapy	2 (3.2)
Masters (MS, MPH, MHA, MEd, MA, MBA)	22 (33.9)
Medical Doctor (MD/DO)	1 (1.6)
Nurse Practitioner/Physician Assistant	1 (1.6)
None	27 (43.5)
Diplomate Training (missing = 1)	
Orthopedics	5 (8.1)
Rehabilitation	4 (6.5)
Acupuncture	3 (4.8)
Sports Medicine	3 (4.8)
Other	5 (8.1)
None	41(66.1)
Prior Hospital Based Training (Student/Resident)	25 (40.3)
Prior Interdisciplinary Employment	37 (59.7)
Chiropractic College Academic Affiliation	34 (54.8)
Medical College Academic Affiliation (missing = 1)	5 (8.2)

*SD* Standard Deviation

Where not otherwise indicated, there was no missing data

### Consultation request

Table 2 summarizes frequency and characteristics of VA chiropractic consultation requests. The majority of respondents (*n* = 55, 88.7%) indicated they had been referred a patient with a prior lumbar fusion within the past month, and most (*n* = 56, 90.3%) had evaluated or treated at least 1 post-lumbar fusion patient in the last month. Two-thirds (*n* = 41, 66.1%) reported seeing between 1 and 5 post-lumbar fusion patients in the past month. Reasons for patient consultations were most commonly for chronic pain (*n* = 60, 96.8%) and/or poor mobility (*n* = 38, 61.3%). Requests were most commonly received from providers in primary care (*n* = 57, 91.9%), physical medicine and rehabilitation (*n* = 40, 64.5%), and neurological/orthopedic surgery (*n* = 36, 58.1%) departments.

**Table 2:** Consultation Requests (n = 62)

Characteristic	Mean (SD) or Number (%)
Number of Patients, whom received fusion surgical procedure performed within the last 6 months, in a typical month (Missing = 1)	
0	20 (32.8)
1–5	30 (49.2)
6–10	6 (9.8)
11–15	5 (8.2)
Number of Post Fusion Referrals in Past Month	
0	7 (11.3)
1–5	39 (62.9)
6–10	14 (22.6)
11–15	2 (3.2)
Number of Post Fusion Patients Examined/Treated in Past Month	
0	6 (9.7)
1–5	41 (66.1)
6–10	10 (16.1)
11–15	3 (4.8)
16–20	1 (1.6)
≥ 21	1 (1.6)
Local Policy Limitation	
No	53 (85.5)
Yes	0 (0)
Unsure	9 (14.5)
Is more than 1 DC reviewing/accepting consults?	
Yes	28 (45.2)
No	34 (54.8)
Number of Providers Triaging Consult Request	1.8 (SD 0.9)
Referring Departments^a^	
Neuro/Orthopedic Surgery	36 (58.1)
Pain Management	6 (9.7)
Physical Medicine and Rehabilitation	40 (64.5)
Physical/Occupational Therapy	15 (24.2)
Primary Care	57 (91.9)
Other (Emergency, Neurology, Direct Patient)	10(16.1)
Reason for Referral^a^	
Chronic Pain	60 (96.8)
Neurological Deficit	13 (21.0)
Poor Mobility/Function	38 (61.3)
Postsurgical Care (Immediately Following Procedure)	1 (1.6)
Other	4 (6.5)

^a^Referrals may have come from more than one source or had more than one reason

### Views on patient evaluation and clinical decision making

**Fig. 1:**
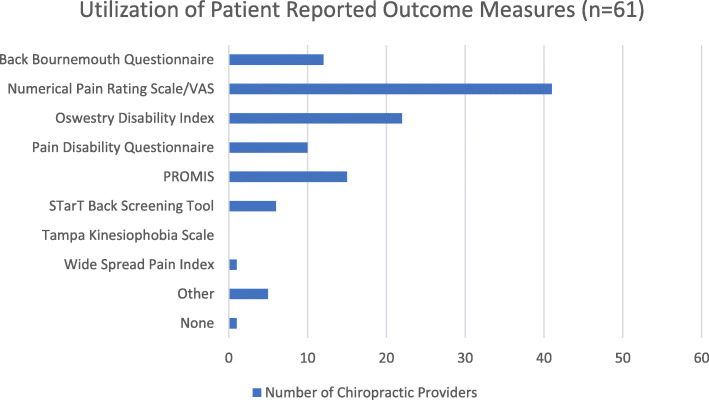
Patient-Reported Outcome Measure Utilization

Table 3 gives an overview of the attitudes and beliefs of VA DCs regarding evaluation and clinical management. Fifty-eight (93.5%) respondents indicated that 12 chiropractic sessions or less were sufficient dosage for a post-lumbar fusion patient to reach MTB. Thirty-three (55%) VA DCs required plain film radiographs with AP, lateral and oblique views, and 21 (35%) required flexion-extension views before considering a trial of care. Ten (16.7%) respondents indicated no specific imaging requirements, and 7 (11.7%) indicated the need to see advanced imaging before the initiation of a trial. In the current study, utilization of patient-reported outcome measures was variable (Fig. 1). Numerical rating scale (*n* = 41, 67.2%), Oswestry Disability Index (n = 22, 36.1%), Patient-Reported Outcomes Measurement Information System (PROMIS) (*n* = 15, 24.6%), and Back Bournemouth Questionnaire (*n* = 12, 19.7%) were the most routinely used tools.

**Table 3:** Evaluation and Clinical Decision Making

Characteristic	Mean (SD) or Number (%)
Appropriate Number of Treatments to Reach MTB	
1–3	1 (1.6)
4–6	7 (11.3)
7–9	24 (38.7)
10–12	26 (41.9)
≥ 13	4 (6.5)
Typical Imaging Requirements for Patients with Prior Lumbar Fusion (Missing = 2)	
No Specific Requirement	10 (16.7)
Lumbar Radiograph (AP/Lateral/Oblique)	33 (55)
Lumbar Radiograph (Flexion/Extension)	21 (35)
CT Scan	1 (1.7)
MRI with Contrast	6 (10)
Earliest Initiation of Thrust Manipulation Following L4–5 Fusion Surgery (Lumbar Region) (Missing = 9)	
1 Month	0 (0.0)
6 Months	11 (20.8)
1 Year	25 (47.2)
≥ 2 Years	4 (7.5)
Never	13 (24.5)
Earliest Initiation of Thrust Manipulation Following L4–5 Fusion Surgery (Thoracic and/or Pelvic Regions) (Missing = 12)	
1 Month	5 (10.0)
6 Months	32 (64.0)
1 Year	12 (24.0)
≥ 2 Years	0 (0)
Never	1 (0.2)
Educational Materials Regularly Provided to Patient (Missing = 1)	
Written Materials	16 (26.2)
Online Resources	10 (16.4)
Not Regularly Provided	35 (57.4)
Communication with Spinal Surgeon for post fusion patients (Missing = 1)	
I don’t receive/accept referrals for post fusion patients	5 (8.2)
1 time per day	0 (0)
1 time per week	1 (1.6)
1 time per month	11 (18.0)
1 time per year	15 (24.6)
< 1 time per year	29 (47.5)
Communication with Referring Provider for post fusion patients (Missing = 1)	
I don’t receive/accept referrals for this population	3 (4.9)
1 time per day	0 (0)
1 time per week	4 (6.6)
1 time per month	30 (49.2)
1 time per year	15 (24.6)
< 1 time per year	9 (14.8)

*MMI* Maximum Therapeutic Benefit

Slightly more than two-thirds of the respondents (n = 36, 67.9%) were willing to initiate a trial of lumbar thrust manipulation 1-year post fusion operation, and one-quarter (*n* = 13, 24.5%) were not willing to initiate thrust manipulation ever following surgical fusion. Three-quarters (*n* = 37, 74.0%) of VA DCs would initiate thrust manipulation to the thoracic or pelvic region at 6 months post-operation, and nearly all of them (n = 49, 98%) would initiate by 1-year post-operative.

A majority of respondents (*n* = 44, 72.1%) indicated that they communicate with the surgical provider 1 time a year or less. More than half (*n* = 34, 55.7%) interacted with the referring provider (potentially including surgical providers) on a weekly or monthly basis, and a minority (n = 9, 14.8%) communicate to the referring provider less than 1 time per year.

### Post-lumbar fusion therapeutic approaches

Table 4 summarizes the reported frequencies of chosen intervention strategies by VA DCs for patients who were post-lumbar fusion. Healthy lifestyle advice (*n* = 56, 94.9%), chronic pain education (n = 53, 89.8%), exercise prescription (*n* = 52, 81.3%), and soft tissue manual therapy [muscle stretching (n = 36, 66.1%), myofascial therapy (*n* = 37, 62.7%)] were the most common approaches selected. More than half of the surveyed DCs (n = 34, 57.6%) indicated the use of thrust spinal manipulation to the thoracic region on an *always* or *frequent* basis for patients post-lumbar fusion. Substantially less respondents indicated the use of thrust manipulation to the pelvic (*n* = 23, 39%) and lumbar (*n* = 10, 17%) regions on an always or frequent basis for patients following lumbar fusion. Spinal mobilization and flexion-distraction manipulation were commonly incorporated treatments selected on an *always or frequent* basis. Lumbopelvic/abdominal stabilization exercise (n = 38, 64.4%) prescription was more commonly utilized (selected *always or frequently*) than Mechanical Diagnosis and Therapy (McKenzie Method) (*n* = 14, 23.7%) and neurodynamic mobilization (*n* = 10, 16.9%). The least common approaches (selected always or frequently) included pelvic blocking (*n* = 0, 0%), and mindfulness meditation (*n* = 6, 10.2%).

**Table 4:** Frequency of Therapeutic Approaches by VA Chiropractors for Patients with Prior Lumbar Fusion Surgery (%) (*n* = 59)

Intervention	Always	Frequently	Sometimes	Rarely	Never
Patient History	59 (100)	0 (0)	0 (0)	0 (0)	0 (0)
Physical Examination	59 (100)	0 (0)	0 (0)	0 (0)	0 (0)
Healthy Lifestyle Advice	41 (69.5)	15 (25.4)	2 (3.4)	1 (1.7)	0 (0)
Education on chronic pain and/or pain neurophysiology	37 (62.7)	16 (27.1)	6 (10.2)	0 (0)	0 (0)
Mobilization	14 (23.7)	19 (32.2)	20 (33.9)	6 (10.2)	0 (0)
Flexion-Distraction	11 (18.6)	21 (35.6)	9 (15.3)	4 (6.8)	14 (23.7)
Pelvic Blocking	0 (0)	0 (0)	8 (13.8)	12 (22.4)	37 (63.8)
Acupuncture/Dry Needling	1 (1.7)	19 (32.2)	12 (20.3)	1 (1.7)	26 (44.1)
Thoracic HVLA	5 (8.5)	29 (49.2)	24 (40.7)	0 (0)	1 (1.7)
Lumbar HVLA	0 (0)	10 (16.9)	17 (28.8)	17 (28.8)	15 (25.4)
Pelvic HVLA	3 (5.1)	20 (33.9)	20 (33.9)	9 (15.3)	7 (11.9)
Muscle Stretches	11 (18.6)	28 (47.5)	14 (23.7)	4 (6.8)	2 (3.4)
Myofascial Therapy (Instrument or Manual)	6 (10.2)	31 (52.5)	14 (23.7)	4 (6.8)	4 (6.8)
Mechanical Diagnosis and Therapy (McKenzie)	5 (8.5)	9 (15.3)	16 (27.1)	12 (20.3)	17 (28.8)
Lumbopelvic/Abdominal Stabilization Exercises	10 (16.9)	28 (47.5)	15 (25.4)	5 (8.5)	1 (1.7)
Neurodynamic Mobilizations	1 (1.7)	9 (15.3)	18 (30.5)	11 (18.6)	20 (33.9)
Cognitive Behavioral Therapy	1 (1.7)	13 (22.0)	18 (30.5)	7 (11.9)	20 (33.9)
Mindfulness Meditation	0 (0)	6 (10.3)	17 (29.3)	19 (32.8)	16 (27.6)
Instrument Assisted Manipulation (Activator, etc.…)	3 (5.2)	13 (22.4)	12 (20.7)	7 (12.1)	23 (39.7)
Advice General Condition and Physical Activity	36 (61.0)	21 (35.6)	2 (3.4)	0 (0)	0 (0)
Specific Exercise Recommendations	25 (42.4)	27 (45.8)	5 (8.5)	2 (3.4)	0 (0)

Write-in interventions: passive modalities (*n* = 3), Proprioceptive Taping (*n* = 1), Home self-traction (*n* = 1), Aquatic Therapy (*n* = 1), Complementary and Natural Medicine (*n* = 1)

HVLA = High Velocity, Low Amplitude Manipulation

## Discussion

Our study presents the first provider-level data on the management of patients with prior lumbar fusion by VA chiropractors. Veterans with a history of lumbar fusion commonly present in VA chiropractic clinics. The majority of respondents described treating patients with prior lumbar fusion within the past month. Survey responses suggest heterogeneity amongst VA chiropractors in the approach to the management of patients with a history of lumbar fusion. Although we are not aware of any published epidemiological data related to the prevalence of lumbar fusion presenting to VA chiropractic clinics, several case reports describe fusion patients seeking chiropractic care in the US [21–25]. It may therefore not be surprising that a large percentage of VA DCs reported managing Veterans with prior lumbar fusion in the last month.

Referrals were primarily to address chronic pain and poor mobility, which was consistent with results of a prior VA DC provider survey describing general referrals [38]. Most of the referrals in the current study were from primary care, physical medicine and rehabilitation, or neurological/orthopedic surgery departments. In the general US population chiropractors are direct access providers, however in the VA system all patients are referred by primary care or specialty service lines. The National Board of Chiropractic Examiners reports that 90.8% of chiropractors work in chiropractic offices, whereas 7.8% are employed in integrated health care facilities, such as VA [39].

Imaging and patient-reported outcome measures (PROMs) may play a role in clinical decision making. Most respondents in the current study require lumbar radiographs with or without flexion-extension views prior to initiating treatment for patients with prior lumbar fusion. Plain film radiographs are the most commonly utilized imaging after fusion due to ease of accessibility, low expense and relatively low-level of radiation exposure [40]. Addition of flexion-extension views has the benefit of allowing for assessment of stability at the fusion site and integrity of related hardware [41]. VA chiropractors frequently incorporated PROMs as is recommended by the American Academy of Orthopedic Surgeons [42], North American Spine Society [43] and Council of Chiropractic Guidelines and Practice Parameters (CCGPP) [44]. PROMS can be useful for allowing providers to objectively measure outcomes for individual patients, and to analyze findings for groups of patients, without the subjectivity of general provider impressions.

Following lumbar fusion, most respondents were of the opinion that the typical dosage for a chiropractic trial to reach MTB was 12 sessions or less. This was in agreement with a chiropractic care pathway for Veterans with low back pain described in 2018 [45], and a 2016 clinical practice guideline for chiropractic care of low back pain from the CCGPP [44]. Neither the care pathway for Veterans nor clinical practice guideline are specific to patients with prior fusion. However, the treatment dosage reported by VA chiropractors for patients who present with added complexity of a prior surgical fusion appears to be similar to each of these guidelines for acute and chronic low back pain [44, 45]. Randomized trials specific to the postsurgical population are needed to inform clinical guidelines specific to post-fusion chiropractic care.

In the current study, several providers indicated they make management and intervention determinations for patients with prior lumbar fusion on an individualized basis, and with additional caution. This appeared to be consistent with the large variety of therapeutic approaches reported by respondents and a paucity of treatments selected as *always* applied. Despite chiropractic providers commonly being associated with thrust-type manipulative therapy, less than half of the providers reported *frequently or sometimes* providing thrust-type manipulation to the region immediately surrounding the fusion (lumbar). The management strategies and intervention techniques commonly chosen as *always* or *frequently* included healthy lifestyle advice, pain education, exercise prescription, “non-thrust” manual therapies, and away from manipulative therapy, particularly with regard to the fused lumbar region. Distraction manipulation and mobilization were among the most commonly described manual treatments [26]. This differed somewhat from a survey of chiropractic treatments by Clijsters et al. which identified the preferred treatments for lumbar conditions for patients without prior surgery as diversified (thrust) manipulation, followed by drop-table assisted and instrument-assisted manipulative techniques [46]. Our study varied from results of a therapist-reported survey describing outpatient physical therapy after spinal fusions which focused on advice/reassurance (98%), instructing active patient treatments such as home exercise programs (91%), abdominal muscle exercises (83%), lumbosacral stabilization exercise (83%), back muscle endurance exercises (76%), and the passive treatments neurodynamic mobilizations (74%) and muscle stretches (67%) [30].

To date there are no published systematic reviews that evaluate chiropractic or spinal manipulation after lumbar fusion [28]. A systematic review of physical therapy and rehabilitation treatments identified good evidence to recommend patient education as a part of pre- and post-operative fusion rehabilitation, moderate evidence to recommend neutral spine control exercise to increase core strengthening as part of operative fusion rehabilitation, and moderate evidence for adding postoperative psychological coping techniques to a rehabilitation program [47]. There is insufficient evidence to recommend joint mobilization, nerve mobilization, and soft-tissue mobilization.

This was only the second survey comprised solely of VA chiropractic providers that has been performed [38]. Our provider response rate of 46.2% was slightly more than half the response rate (91.6%) of the former study published in 2009 [38], but our questionnaire (n = 62) surveyed nearly twice as many providers as the 2009 study (n = 36). The VA has seen substantial growth in the number of employed chiropractic providers (21.3% annually) since the prior survey was performed and it is reasonable that this increase in providers has led to the reduced response rate [17].

Our survey response rate was comparable with other self-administered surveys of physicians. In a meta-analysis of response rates of 45 web-based surveys the mean response rate was 39.6% [48], and another study specifically exploring physician specialist response rates to web-based surveys had an overall survey response rate of 35.0%. Cunningham et al. described a wide variation in response rates by specialty, with neurology/neurosurgery (46.6%) being the highest and psychiatry (27.1%) being the lowest [49]. Results from other studies which surveyed DC provider cohorts had similar responses to ours. A 2017 survey of chiropractic radiologist had a response rate of 38.4% [50], and a 2018 study of chiropractic practitioners had a response rate of 43.2% [51]. To our knowledge, this is the first study to survey any cohort of the chiropractic profession regarding the care of a patient with prior lumbar fusion, or any prior lumbar surgical procedure. Assessment of patient outcomes was beyond the scope of this project, however this should be explored in future work.

Our study appeared to be a good representation of VA chiropractic field in regards to age, gender, and years of experience. A cross-sectional analysis of VA administrative data in 2016 indicated that the typical VA chiropractor employee was a 45.9-years-old, male (81.4%) and had worked at VA for 4.5 years [17]. This closely matched our respondents’ population mean age of 44.5 years, gender (80.6% male), and the majority were employed at VA less than 6 years (74.7%).

### Limitations

There were several limitations to our survey. Although this was a national sample of all VA-employed chiropractors, the limited sample size may result in some findings being under- or over-represented. There may be selection bias due to the response rate (46.2%), as providers less likely to agree with study findings may have chosen not to respond. We did not attempt to account for differences in approach by geographic or respondent demographic factors. Several questions relied on self-report and not patient records, which could be subject to retrospective recall bias. Further, this study included the attitudes and beliefs of VA DCs related to the management of patients with prior lumbar fusion and may not be generalizable to the chiropractic profession in general. Likert categories utilized in the instrument matrix table were open to practitioner interpretation and may not have been interpreted in the same way by all respondents. Several treatment approaches were not provided as multiple-choice options and were written in by participants, and thus may have been underreported. Respondents specifically mentioned the use of drop table assisted techniques, passive modalities (e.g. electrical stimulation, therapeutic ultrasound, etc...), proprioceptive taping, and education/distribution of durable medical equipment (e.g. braces, self-traction devices, etc...). We did not include physical modalities, taping and/or equipment prescription in the survey instrument because, due to a paucity of definitive evidence, they are not recommended by CCGPP for routine low back pain [44].

## Conclusion

This survey provides preliminary data regarding chiropractic management approaches to patients with prior lumbar fusion among VA chiropractors. There does not appear to be a consensus on treatment strategies for this population. Most providers seemed to employ chronic pain education, spinal manipulation to non-fused regions (thoracic, pelvic), “non-thrust” manual therapies for the lumbar region and/or rehabilitative exercise. Patients with lumbar fusion regularly present for chiropractic care and the data support the need for continued study of management practices for this population.

## Data Availability

Access to the data will be made upon request from the corresponding author.

## Abbreviations

CCGPP: Council of Chiropractic Guidelines Practices and Parameters
DC: Doctor(s) of Chiropractic
LBP: Low Back Pain
MTB: Maximum Therapeutic Benefit
US: United States
VA: Veterans Affairs

## References

[1] Hoy D, March L, Brooks P (2014). The global burden of low back pain: estimates from the Global Burden of Disease 2010 study. Ann Rheum Dis.

[2] Deyo RA (2015). Fusion surgery for lumbar degenerative disc disease: still more questions than answers. Spine J.

[3] HCUPnet. Agency for Healthcare Research and Quality. Available at: http://hcupnet.ahrq.gov/HCUPnet.jsp. Accessed May 19, 2016.

[4] Martin BI, Mirza SK, Spina N (2019). Trends in lumbar fusion procedure rates and associated hospital costs for degenerative spinal diseases in the United States, 2004 to 2015. Spine (Phila Pa 1976).

[5] Carragee EJ, Han MY, Suen PW, Kim D (2003). Clinical outcomes after lumbar discectomy for sciatica: the effects of fragment type and annual competence. J Bone Joint Surg Am..

[6] Maigne JY, Planchon CA (2005). Sacroiliac joint pain after lumbar fusion. a study with anesthetic blocks. Eur Spine J.

[7] Fritsch EQ, Heisel J, Rupp S (1996). The failed back surgery syndrome: reasons, intraoperative findings, and long-term results: a report of 182 operative treatments. Spine.

[8] Walker BF, Hebert JJ, Stomski NJ (2013). Short-term usual chiropractic care for spinal pain. Spine (Phila Pa 1976).

[9] Goertz CM, Long CR, Hondras MA (2013). Adding chiropractic manipulative therapy to standard medical care with patients with acute low back pain: results of a pragmatic randomized comparative effectiveness study. Spine (Phila PA 1976).

[10] Haas M, Vavrek D, Peterson D (2014). Dose-response and efficacy of spinal manipulation for care of chronic low back pain: a randomized controlled trial. Spine J.

[11] Bronfort G, Hondras MA, Schulz CA (2014). Spinal manipulation with home exercise with advice for subacute and chronic back-related leg pain. Ann Intern Med.

[12] Goertz CM, Long CR, Vining RD (2018). Effect of usual medical care plus chiropractic care vs usual medical care alone on pain and disability among US service members with low back pain: a comparative effectiveness clinical trial. JAMA Network Open.

[13] Qaseem A, Wilt TJ, McLean RM, Forciea MA (2017). Noninvasive treatments for acute, subacute, and chronic low back pain: A clinical practice guidelines from the American College of Physicians. Ann Intern Med.

[14] Barnes PM, Powell-Griner E, McFann K (2002). Complementary and alternative medicine use among adults: United States. Adv Data.

[15] Barnes PM, Bloom B, Nahin RL (2007). Complementary and alternative medicine use among adults and children: United States. Natl Health Stat Rep.

[16] Heward J, Jones CM, Compton WM (2018). Coverage of nonpharmacologic treatment for low back pain among US public and private insurers. JAMA Open Network.

[17] Lisi AJ, Brandt CA (2016). Trends in the use and characteristics of chiropractic services in the Department of Veterans Affairs. J Manipulative Phyisol Ther.

[18] Apegren DD, Burt AL (1994). A study of postspinal surgery cases in chiropractic offices. J Manipulative Physiol Ther.

[19] Hurwitz EL, Coulter ID, Adams AH (1998). Use of chiropractic services from1985 through 1991 in the United States and Canada. Am J Pub Health.

[20] Stern PJ, Cote P, Cassidy JD (1995). A series of consecutive cases of low back pain with radiating leg pain treated by chiropractors. J Manipulative Physiol Ther.

[21] Kruse RA, Cambron J (2011). Chiropractic management of postsurgical lumbar spine pain: a retrospective study of 32 cases. J Manipulative Physiol Ther.

[22] Kruse RA, Cambron J (2011). Cox decompression chiropractic manipulation of a patient with postsurgical lumbar fusion: a case report. J Chiropr Med.

[23] Greenwood DM (2012). Improvement in chronic low back pain in an aviation crash survivor with adjacent segment disease following flexion distraction therapy: a case study. J Chiropr Med..

[24] McGregor M, Cassidy JD (1983). Post-surgical sacroiliac joint syndrome. J Manipulative Physiol Ther.

[25] Morningstar MW, Struchman MN (2012). Manipulation under anesthesia for patients with failed back surgery: retrospective report of 3 cases with 1-year follow-up. J Chiropr Med.

[26] Gudavalli MR, Olding K, Joachim G, Cox JM (2016). Chiropractic distraction spinal manipulation on postsurgical continued low back and radicular pain patients: a retrospective case series. J Chiropr Med.

[27] Perrucci R, Coulis C (2017). Chiropractic management of post spinal cord stimulator spine pain: a case report. Chiropr Man Ther.

[28] Daniels CJ, Wakefield PJ, Bub GA, Toombs JD (2016). A narrative review of lumbar fusion surgery with relevance to the chiropractor. J Chiropr Med.

[29] Becker WC, DeBar LL, Heapy AA (2018). A research agenda for advancing non-pharmacological management of chronic musculoskeletal pain: Findings from a State-of-the-art conference. J Gen Intern Med.

[30] Rushton A, Wright C, Heap A (2014). Survey of current physiotherapy practice for patients undergoing lumbar spinal fusion in the United Kingdom. Spine (Phila Pa 1976).

[31] Burns KE, Duffett M, Kho ME (2008). A guide for the design and conduct of self-administered surveys of clinicians. CMAJ.

[32] Drauglais JR, Coons SJ, Plaze CM (2008). Best practices for survey research reports: a synopsis for authors and reviewers. Am J Pharm Educ.

[33] Kelly K, Clark B, Brown V (2003). Good practice in the conduct and reporting of survey research. Int J Qual Health Care.

[34] von Elm E, Altman DG, Egger M (2008). The Strengthening the Reporting of Observational Studies in Epidemiology (STROBE) statement: guidelines for reporting observational studies. J Clin Epidemiol.

[35] Harris PA, Taylor R, Thielke R (2009). Research 430 electronic data capture (REDCap) – A metadata driven methodology and workflow process for providing translational research informatics support. J Biomed Inform.

[36] Harris PA, Taylor R, Minor BL (2019). The REDCap consortium: building an international community of software partners. J Biomed Inform.

[37] U.S. Department of Veterans Affairs. Chiropractic care facility locations. https://www.rehab.va.gov/chiro/locations.asp. Accessed March 10, 2020.

[38] Lisi AJ, Goertz C, Lawrence DJ, Satyanarayana P (2009). Characteristics of Veterans Health Administration chiropractors and chiropractic clinics. J Rehabil Res Dev.

[39] Christensen MG, Hyland JK, Goertz CM, Kollasch WM (2015). The Chiropractic Practitioner. Practice Analysis of Chiropractic.

[40] Venu V, Vertinsky AT, Malfair D (2011). Plain radiograph assessment of spinal hardware. Semin Musculoskelet Radiol.

[41] Ray CD (1997). Threaded fusion cages for lumbar interbody fusions: an economic comparison with 360 degree fusions. Spine (Phila Pa 1976).

[42] American Academy of Orthopaedic Surgeons. Patient reported outcome measures. https://www5.aaos.org/CustomTemplates/landingPage.aspx?id=4294968282&ssopc=1. Accessed March 10, 2020.

[43] Kreiner DS, Hwang SW, Easa JE (2014). An evidence-based clinical guideline for the diagnosis and treatment of lumbar disc herniation with radiculopathy. Spine J.

[44] Globe G, Farabaugh RJ, Hawk C (2016). Clinical practice guideline: chiropractic care for low back pain. J Manipulative Physiol Ther.

[45] Lisi AJ, Salsbury SA, Hawk C (2018). Chiropractic integrated care pathway for low back pain in veterans: results of a Delphi consensus process. J Manipulative Physiol Ther.

[46] Clijsters M, Fronzoni F, Jenkins H (2014). Chiropractic treatment approaches for spinal musculoskeletal conditions: a cross-sectional survey. Chiropr Man Therap.

[47] Madera M, Brady J, McGinty T (2017). The role of physical therapy and rehabilitation after lumbar fusion surgery for degenerative disease: a systematic review. J Neurosurg Spine.

[48] Cook C, Heath F, Thompson R (2000). A meta-analysis of response rates in web- or internet-based survey. Educ Psychol Measurement.

[49] Cunningham CT, Quan H, Hemmelgarn B (2015). Exploring physician specialist response rate to web-based surveys. BMC Medical Research Methodology.

[50] Young KJ (2017). Historical influence on the practice of chiropractic 452 radiology: part 1 – a survey of Diplomates of the American Chiropractic College of Radiology. Chiropr Man Ther.

[51] Taylor DN, Wynd S (2018). Survey of chiropractic clinicians on self-reported knowledge and recognition of concussion injuries. Chiropr Man Ther.

